# Computed Tomography in Craniofacial Fibrous Dysplasia: A Case Series with Review of Literature and Classification Update

**DOI:** 10.2174/1874210601711010384

**Published:** 2017-06-30

**Authors:** Deepak Gupta, Preeti Garg, Amit Mittal

**Affiliations:** 1 Department of Oral Medicine and Radiology, M.M. College of Dental Sciences and Research, Mullana, Ambala, Haryana, India.; 2 Department of Oral Medicine and Radiology, M.M. College of Dental Sciences and Research, Mullana, Ambala, Haryana, India.; 3 Department of Radiodiagnosis and Imaging, M.M. Institute of Medical Sciences and Research, Mullana, Ambala, Haryana, India.

**Keywords:** Classification update, Computed tomography, Craniofacial fibrous dysplasia, Fibrous dysplasia, Ground glass appearance, Panoramic radiographs

## Abstract

**Introduction:**

Fibrous dysplasia (FD) is a fibroosseous lesion of the osseous structures of the body. It is not a commonly reported lesion yet it is considered as an important lesion which can affect the maxillofacial region as well. As a result, it can cause deformity of the jaw bones which can further lead to severe facial asymmetry. Craniofacial fibrous dysplasia (CFD) is one of the subtypes of FD that can affect the bones of the craniofacial complex, including the mandible and maxilla. It can also present as facial asymmetry and can be investigated with the help of Maxillofacial Radiology and Imaging. The radiographic findings may vary according to the extent and degree of the disease. Although conventional radiographs provide a good clue regarding the lesion, advanced maxillofacial imaging is capable of providing detailed extent of the disease. Furthermore the classification of CFD is not very clear in the literature.

**Case Report:**

This particular paper attempts to document and report the CT appearance of CFD with an attempt to propose a better classification system for the same. Four different patients are reported which presented with FD with involvement of bones of craniofacial region. Working diagnosis of CFD was made with the help of clinical features as well as with radiographic assessment. Advanced imaging included CT scan of the lesions. The article highlights the importance of computed tomography in diagnosis as well as assessment of extent of the disease.

**Conclusion:**

It can be concluded that the Dental professionals must be aware regarding the different radiographic appearances of CFD. Advanced imaging modality like CT can provide with exact diagnosis as well as extent of the lesions like FD. Further collaboration of researchers is required to incorporate this proposed change in classification of CFD.

## 1 INTRODUCTION

FD is a benign fibrosseous bone disease or hermartomatous condition of bone [1-15]. It was originally described by Lichtenstein more than sixty years ago [1, 16]. It presents with replacement of the osseous structures with cellular fibrous tissue containing foci of ossification [1-4, 17-20]. Researchers have revealed its association with mutation in the gene (GNAS I) [21-24] which results in the encoding of the subunit of stimulatory G-protein (Gs) [21-24]. This will eventually results in the proliferation and differentiation of preosteoblasts due to increased production of cyclic adenosine monophosphate (cAMP) [2, 4-9, 21-24]. It is evident from the literature that most frequently (in almost 80% of the cases) FD affects only one bone [17-20] and this form is called monostotic FD while in case multiple bones are involved, it is called as polyostotic FD [2, 10, 11]. The polyostotic form can also present features like café-au-lait skin pigmentation and precocious puberty in McCune-Albright syndrome, which affects mainly young females [4, 9, 17-20]. When the sphenoid, zygomatic, frontonasal bones and the base of the skull are affected, the disease is called craniofacial FD [12, 13, 17-20]. Craniofacial involvement in FD is seen in both monostotic and polyostotic forms [9, 10]. Monostotic FD has a different skeletal distribution from polyostotic disease and occurs most commonly in the femur followed by tibia, craniofacial bones, and ribs [14]. Craniofacial involvement occurs in about 30% of monostotic fibrous dysplasia and typically affects the maxilla, mandible, and rarely the calvarium. Polyostotic form of the disease has nearly 100% involvement of the craniofacial bones [15].

It should be noted that literature highlights the term monostotic to be applied to those cases of FD which involved the mandible alone [2, 25, 26]. As per these words, this cannot be true for those cases of FD with the involvement of maxilla because of the presence of contiguous bones like zygoma [2, 25, 26]. These type of cases have been referred to as “CFD” [2, 25, 26]. Henceforth it can be quoted that craniofacial FD is more frequently seen in the maxilla as compared to the mandible [7, 8, 21-24]. It can cause severe deformities and blindness [2, 8]. Bones affected by FD can be treated by surgical remodelling for aesthetics and for functional purposes after the disease becomes dormant [10, 11, 25-27].

Sometimes it is difficult to differentiate FD from other bony lesions like Paget’s disease and cementoosseous fibroma because of the virtually similar radiographic presentation of these lesions [17-20, 28-36]. The Diagnosis becomes further difficult when the lesion has negligible clinical evidence but shows changes during radiographic evaluation. The lesions may even present as extensive lesions in the maxillofacial region [32]. Henceforth Dental professionals should be aware regarding the different radiographic appearances of CFD [28, 32, 37].

Several researchers have highlighted that the functional and aesthetic impairment caused by CFD may incapacitate the affected individual [10, 25, 26]. Further literature also reveals a varied discussion in diagnosis of the same [10, 25, 26] Therefore, we hereby present a couple of cases diagnosed with CFD so as to contribute towards the diagnosis as well as better understanding of this disease process. This article reviews the pertinent clinical as well as radiographic features (Computed Tomographic features) of CFD [10, 25, 26].

### 2 CLINICAL PRESENTATION

Clinical presentation of FD varies with the primary bone involved and the extent of disease [8]. Further it is of interest to know that the extent of this disease has no correlation with the onset of the disease [2, 25]. FD has its onset during early life, usually in late childhood or early adolescence [10]. Patients with polyostotic form of disease are reported to be considerably younger. Literature reveals that this disease usually presents during the initial three decades of life with markedly progressive course with varying clinical features [10, 25, 26]. However it is still not uncommon for the disease to resume continued progression in later stages even after the 3^rd^ decade [10, 25, 26]. There is an equal sex distribution in monostotic FD but the polyostotic form has been reported to have a clear female predilection [8].

CFD usually presents as a painless swelling [10, 25, 26, 38]. Other presenting signs and symptoms depends upon the area of involvement [16-20]. The progression of the disease stops by itself after the maturation of the craniofacial skeleton [4, 10, 25, 26, 38]. CFD may even lead to certain dreaded conditions like loss of vision or other optic disorders [16-20] which may include loss of colour vision, peripheral/central field defects, and afferent pupillary defect [10, 25, 26, 38]. It is attributed to stenosis of the optic canal resulting in reduced retinal perfusion [10, 25, 26, 38]. Involvement of Sphenoid and ethmoid bones by this disease may also lead to globe displacement of the eyeball [7, 26, 38]. Visual impairment once noted in the patient is progressive with periods of exacerbation and remission [10, 25, 26, 38]. Literature also reveals the evidence of involvement of temporal bone in CFD leading to hearing loss as a result of stenosis of the external auditory canal [10, 19, 25, 26].

Involvement of Frontal, sphenoid, nasoethmoid, and maxillary bones may result in nasal obstruction, sino-ostial obliteration and subsequent sinusitis [10, 25, 26]. Other features associated with Cranial base and orbital involvement are dystopia, dysesthesias in the distribution of the trigeminal nerve, epiphora and headaches [10, 19, 20, 25, 26]. Bones affected by CFD may also lead to cystic degeneration and other cystic lesions such as aneurysmal bone cysts [32].

Further, FD may rarely present with unerupted teeth and root resorption [9, 19, 20]. On the contrary displacement of the teeth may be evident. The teeth may be displaced even into the maxillary sinus with the fibro-osseous tissue when maxillary sinus is involved [9, 19, 20].

## 3 DIAGNOSIS

Although histopathology is considered as a gold standard to diagnose any disease entity, radiography particularly CT in CFD has got a significant role in diagnosis, treatment planning as well as in follow up. Many authors in the literature have supported the above said fact [1-15]. Literature also reveals that the margins, internal structure, and effect on adjascent structures in FD are best characterized on CT images [3, 39-44]. Several lesions like CGCG (Central giant cell granuloma), ameloblasitic fibroma, ossifying fibroma etc. may present with similar radiographic presentation like CFD [9, 19, 20]. Authors reveal that the density and trabecular pattern of bone in CFD lesions may vary [9, 19, 20] due to combination of osseous as well as fibrous elements [10, 25, 26]. The lesions may be more radiolucent at initial stages which may present as a lytic pattern of trabeculae [10, 24-26]. At later stages it becomes radiopaque as the lesion matures which may present as a sclerotic type [10, 25, 26].

Literature highlights that the most common pattern observed is mixed type with an incidence of 40% while sclerotic type constitutes 35% of reported cases [10, 25]. Skull base is the most frequent anatomical place for this to occur. Lytic pattern is the least common pattern observed[10, 35]. This can be attributed to the fact that most of the times the patient never report during the initial stages of the disease.

Mature lesions in rare cases may appear to have granular internal septa leading to a multilocular appearance [2, 26] of the lesion. The bone affected by CFD may present short, thin and/or irregularly placed trabeculae. Further they can be numerous as compared to trabeculae of normal bone [9, 19, 20, 23]. This will lead to a varied radiographic pattern which may be granular, peau d’orange, whispy (cotton wool), amorphous dense pattern and fingerprint pattern [9, 19, 20, 33, 34]. The granular pattern is moreover an appearance of ground-glass resembling small fragments of shattered windshield [9].

The radiographic picture of FD varies with the internal morphology of the lesion as well as with the anatomic site involved. Craniofacial skeleton consists of thin bones such as orbital plate of maxillary, frontal and ethmoid bones; as well as thick bones like sphenoid and mandible [10]. Thin bones lead to a fast and greater expansion of cortex as compared to thick bones [38, 45]. Henceforth thicker bones when affected with FD display less radiolucency, cavitation, and compartmentalization as compared to thin bones. However, on the contrary, localized FD on MR imaging often mimics a tumor. This is said to be because of fibrous tissue which can enhance brilliantly after the injection of contrast material [3, 17, 18].

It is a well known fact that the facial skeleton is anatomically complex consisting of different bones.Further it is already stated that FD may present as grossly sclerotic and/or thickened lesions [10]. This may lead to overlapping structures if conventional radiology is used in maxillofacial skeleton. Henceforth this may preclude adequate assessment of the lesion with conventional radiography [10, 25, 26]. Thus, CT is considered as a better radiological tool for assessing such lesions.

It is also an important fact that the involvement of optic canals, orbital fissures, frontonasal ducts, and ostiomeatal complex can be best evaluated by CT scanning. Henceforth, it is considered invaluable in pre-operative planning as well as a superior diagnostic tool for follow up in cases of CFD [1, 2, 16, 39].

CT representation of FD is characteristic and consists of three varieties: ground-glass pattern (56%), homogeneously dense pattern (23%) and cystic variety (21%) [ 7, 8, 17, 18, 41]. CT characteristics of FD include expansion of the involved bone with heterogenous pattern of CT densities associated with scattered or confluent islands of bone formation [38, 45]. Depending upon the bony and fibrous content of the lesion, the researchers have reported the CT attenuation to be in the range from 34 to 513 HU [8, 9, 28, 37].

The introduction of 3-dimensional (3D) CT has further helped to improve the localization and visualization of pathology. It also assists in accurate surgical planning as it helps to delineate the exact extent of the lesions [10, 25, 26]. Furthermore CT is less expensive and more available than magnetic resonance imaging (MRI) [10]. However MRI offers greater specificity in neurovascular and ocular involvement and in detection of other soft tissue lesions [10, 25, 26] the usage of MRI in CFD is limited because of reduced signal intensity in such lesions.

Follow up several authors and researchers like Posnick [41] and Costello [42] has recommended a life- long and continuous follow-up for FD [9,19] in case a surgical procedure is performed. This is in order to know that there is no progression of any residual lesion at any stage postoperatively [9, 19, 20]. Cases of CFD must also be monitored for disease activity and progression with a particular emphasis on the thorough assessment of the cranial nerves including visual field testing [10].

### 4 CASE REPORTS

**4.1. Case 1:** A 21 years old brown male was referred to our service for the diagnosis of diffuse asymmetry of the face by a private dental practitioner. Extra-oral examination revealed asymmetry on the left side of the face involving the maxilla, mandible, frontal and temporal bone (Fig.**1A**). On the affected side, an intraoral swelling was detected on the hard palate crossing the midline, which was covered with normal mucosa and was hard on palpation. The patient reported no speech or swallowing problems and the disease was asymptomatic. Patient was subjected to conventional radiographs (Fig. **1B**) including submentovertex view which revealed expansion of the left side of mandible.

On computed tomography with bone window (Figs. **1C**-**1E**), there was evidence of spongy bone with ground glass appearance of bone with lytic regions. The lesion involved the mandible of left side with erosions and destruction of cortical borders at multiple places. Further there was evidence of involvement of temporal, zygomatic, frontal and ethmoid bones (Fig. **1C**). Lesion was crossing the midline in frontal region (Fig. **1D**). Sphenoid bone was also involved with narrowing of the optic canal as well as orbital fissures (Fig. **1C**). Crista Gali was also involved with erosion and destruction in the area of base of skull in the middle cranial fossa. Pterygiod plates of left side were also involved. Tooth displacement was observed towards buccal as well as lingual direction inferiorly. Inferior alveolar nerve canal was displaced inferiorly.

Histopathological analysis revealed the replacement of normal bone with cellular fibrous tissue consisting of spindle- shaped fibroblasts embedded in a moderate amount of collagen. The microscopic diagnosis was “benign fibro-osseous disease.” On the basis of the clinical, radiographic, and microscopic data, a diagnosis of craniofacial FD was established. Patient was subjected to surgical treatment and was followed for years, with clinical and radiologic exams being performed at 3 to 6 month intervals. The FD remained unchanged.

**4.2. Case 2**: A 42 years old male patient reported with asymmetry of the maxillofacial region along with restricted mouth opening since 2 years. The problem started with slight swelling on the right side of the face (Fig. **2A**). Gradually it led to restricted mouth opening. It was progressive in nature. Clinically there was reduced mouth opening with deviation towards the right side while opening. On general physical examination there was evidence of swelling along the left shin of tibia (Fig. **2B**). Working diagnosis of Polyostotic craniofacial FD was made.

The initial screening involved conventional radiography including PNS and Submentovertex view which showed expansion of the right side of the craniofacial structures including maxilla, mandible and temporal bones (Figs. **2C**, **2D**). Lateral AP view of Tibia showed evidence of sclerosis of upper and mid shaft of tibia. Also there were lytic areas in the sclerotic irregularity of the bone (Fig. **2E**).

CT scan showed the involvement of right side of mandible with the TMJ, glenoid fossa, temporal bone, frontal bone and maxilla with ground glass appearance of bone (Figs. **2F**-**2I**). The maxillary sinus of right side was deviated antero-superiorly. Further the zygomatic arch, zygoma, base of the cranium, mastoid process, pterygoid plates and sphenoid of right side were also involved. There was evidence of involvement of occipital condyles of both the sides and clivus. Furthermore there was evidence of a cystic lesion in the right maxilla along with mandible extending from body of the mandible to the ramus of right side (Figs **2H**, **2I**). Aspiration of the lesion was carried out which was suggestive of secondary Aneurysmal bone cyst.

**4.3. Case 3:** A 38-years old male presented with maxillofacial asymmetry. The CT scan showed the involvement of right side of the maxilla including the maxillary sinus, right zygoma, right sphenoid, zygomatic arch, pterygoid plates of right side along with the cranial base with ground glass appearance of bone (Figs. **3A**-**3D**). Further there was evidence of narrowing of the right side of optic canal (Fig. **3D**). There were no other bones involved in the entire body. Radiographic diagnosis of CFD was arrived.

**4.4. Case 4**: A 35 years old male presented to us with an asymptomatic intraoral swelling in the left side of the maxilla at the tuberosity region. There was no obvious facial asymmetry. It was not associated with any difficulty in mouth opening or pain. CT scan revealed involvement of maxillary alveolus and tuberosity along with the maxillary sinus and base of the left pterygoid plate with ground glass appearance of bone (Figs. **4A**-**4C**). The TMJ and the ostiomeatal complex were normal (Fig. **4D**).

## 5 DISCUSSION

FD is a fibro-osseous bone disease and can occur anywhere in the long bones, skull and maxillofacial region of the body. In contrast to some fibro-osseous lesions like Cherubism or Focal cemento-osseous dysplasia, it does not exclusively manifests in the maxillofacial region [9, 19, 20, 23, 43].

Researchers have categorized FD into monostotic variety with single bone involvement and polyostotic variety with multiple bone involvement [21-24  ]. CFD is another type of FD which was identified by Daves and Yardley with the involvement of two or more facial and cranial bones [21, 23, 46]. The prevalence of FD as reported by Eversole *et al.* for monostotic type is 74%, for polyostotic type is 13% and for craniofacial type is 13% [21, 23, 46]. It is evident from the literature that the classification of FD has always been debatable in context of craniofacial variety. Many authors have described craniofacial fibrous dyaplasia as polyostotic even when there is no other bone involved. This can be supported by the fact that Eversole *et al.* [47] categorized the craniofacial type of FD as polyostotic because many bones of the craniofacial complex are involved in this variety [22, 24]. Furthermore in this craniofacial complex, all the bones except mandible are separated from each other only by sutures [22**, **24]. On the other hand many authors have described it as monostotic even when two or more bones of the craniofacial region are involved [46].

Some authors however do not classify the craniofacial FD into monostotic or polyostotic form at all. They just regard the FD as CFD per se [48-54]. According to them, CFD is not truly monostotic because of the involvement of multiple adjacent bones of the craniofacial skeleton [22, 24, 48, 49, 54]. Similarly CFD is not even polystotic because in some cases bones outside the craniofacial complex are usually not involved [22, 24, 48, 49, 54]. This may be attributed to the fact that probably these authors consider craniofacial complex as a single unit. These lesions are more frequently seen in the maxilla as in maxilla they can simultaneously cross sutures and enter into the adjacent facial bones.

Henceforth they are termed as craniofacial FD inspite of monostotic or polyostotic as they do not meet the precise criteria for monostotic or polyostotic forms [12, 17, 18, 48]. Henceforth it is proposed that the craniofacial FD involving a single anatomic area with bones involved in continuity which are actually separated by sutures can be regarded as monostotic if there is no other bone involved in the body. On the contrary if two separate anatomic areas of the craniofacial skeleton are involved, it may also be categorised as polyostotic craniofacial FD regardless of the fact that whether any other bone of the body is involved or not. Further if the craniofacial FD crosses the temporomandibular joint suture leading to involvement of mandible and temporal bone, it should be categorized as polyostotic craniofacial FD. According to the present case series, as FD affected the maxilla and adjacent bones, it was termed as craniofacial FD.

It is of interest to know that in most of the cases, the clinical information and radiographic presentation of polyostotic FD is sufficient enough to allow the practitioner to make a diagnosis without the requirement of biopsy [10, 25, 26]. This is in accordance to this presented case series. In contrast, monostotic FD requires a histopathological examination. The treatment of FD is surgical removal of the bone [9]. Further surgical planning is done based upon the radiographic extent of the lesion. As depicted in the conventional radiographs of the present case series, the conventional radiographs lack in the evaluation of the actual extent and number of bones involved because of superimposition on the craniofacial skeleton. This supports the fact that however the treatment of CFD is being done by the time the growth period is over, there are reports of exaggerated growth from the stimulation of the spared dysplastic lesion [9].

Henceforth it is recommended that in such cases the examination must be supplemented with CT as done in this case series. This leads to a more accurate, 3-dimensional representation of the extent of the lesion [9, 16, 37]. It can also serve as a precise baseline study for future comparisons to follow up the case [9, 34]. The CT findings in this case series shows that the base of the skull is the most frequently involved site in the craniofacial FD [9] which is in accordance to various studies in literature [35].

In the base of the skull, sphenoid bone was found to be the most commonly affected bone [9, 35] as seen in case 1, case 2 and case 3 of the present series. It is also reported to often extend to the basilar portion of the occipital bone [9]. In the vault the most common site of occurrence is frontal bone [9, 35] as seen in case 1 and 2 of this case series. Researchers have highlighted that in CFD cases, mixed radiolucent/radiopaque pattern is the dominant pattern while granular pattern is very rare [9, 16, 35, 37].

When the maxilla is involved, it is more difficult to delineate the extent of CFD with conventional radiographs. In these cases, CT imaging is more helpful as it can precisely delineate the extensions into the antrum, orbit and nasal cavity [9, 37]. Further, authors have also revealed that three-dimensional bone reconstruction with helical CT can lead to optimal visualization of the extent of CFD involving the skull base [9, 37]. It is of interest to know that most of the CFD lesions affecting the maxillofacial region are largely unilateral. Hence, a 3-dimensional mirror-image of the normal contralateral side can be made which may improve the likelihood of postoperative symmetry [9, 37].

Literature reveals that CFD is found to be as common as FD of jaws and is more commonly seen in younger age which is in accordance with this case series. Maxilla and frontal bones were most commonly involved which is in accordance with many studies in literature [23, 44]. It is well documented in the literature that when the maxilla is affected by FD, there are high chances that all the other adjacent bones which are separated by sutures such as zygomatic, sphenoid, frontal, and nasal bones might also get affected [21, 23, 41]. This fact was well in accordance with the cases presented in this case series. All the cases presented with Ground glass appearance of the bone.

The expansion evident in FD shows bucco-lingual expansion which can lead to thinning of the cortical plate. In spite of the expansion of the external surface of the bone, it is of interest to note that the affected bone will still retain recognizable anatomical shape [21, 23, 49]. Literature also reveals that the lesion can displace the inferior alveolar canal in all four directions (buccal, lingual, superior, and inferior) depending upon the epicentre of the lesion [21, 23]. This was in accordance to case 1 of this series in which the canal was displaced inferiorly as the epicentre was at superior position to the canal. This is in contrast to the finding of Petrikowski [50] who suggested upward displacement of the canal to be a unique characteristic of FD [21, 23, 28].

Similarly the lesion involving maxilla may show expansion of the external as well as internal surface. The expansion of the internal surface leads to involvement of maxillary sinus further leading to reduced size of the sinus cavity. However, as discussed earlier, the shape still remains recognizable [21, 23]. This is in accordance to this presented case series. In case 1, the anterolateral and posterior wall of maxillary sinus was involved with FD and the size of the left sinus was small as compared to the right side. Similarly in case 2, the right sided maxillary sinus was displaced superiorly with reduction in the size of the maxillary sinus, although the sinus was still recognizable with open osteo-meatel complex. Case 3 also revealed reduction in the size of right maxillary sinus with involvement of the anterolateral and posterolateral walls at the level of zygoma. Still the shape was maintained a part of the fact that the maxillary sinus was displaced posteriorly and medially. Case 4 also revealed reduced size of the left maxillary sinus cavity with involvement of posterior wall and the floor of the maxillary sinus.

This unique presentation can help in differentiating FD from other tumours like ossifying fibroma [21, 23, 49]. Progression of the disease can also lead to complete obliteration of the maxillary sinus and displacement of the floor of orbit. Further certain radiographic features which can be seen on plain films like loss of lamina dura is also unique to FD [10, 25, 26, 50]. There may be impacted teeth like in case 2 which is caused by increased density of bone in the path of eruption [21, 23].Authors say that there is no bucco-lingual displacement of the tooth [21] as is seen in case 3 and case 4. But on the contrary there was buccolingual displacement of the tooth on left side in case 1.

Researchers highlight that Periapical radiographs can also help to determine the internal structure of FD accurately as it is placed closest to the jaw [17-19, 28]. It is also evident in literature that some lesions of FD present with perilesional sclerosis while some do not. The lesion is considered as sharply defined when this perilesional sclerosis is present. Otherwise it will fade into the adjacent normal bone [17-19, 51]. Certain authors like Sherman and Eversole *et al.* intimate that diffuse and poorly defined lesions may be dysplastic in nature [17-19, 51].

Fries in 1957 described these basic radiographic patterns as pagetoid (56%), a mixture of dense and radiolucent areas of fibrosis; sclerotic (23%), mass is homogeneously dense; and cystic (21%), a spherical or ovoid lucency surrounded by a dense boundary [17-19 , 51]. Patients of FD usually present with a large swelling [17, 18]. This can be attributed to the fact that the lesion remains asymptomatic for long span of time [17, 18]. Thus patients suffering from this disease do not seek dental care unless there is gross facial asymmetry or pain due to compression of nerves [17-19, 28]. In this case series, all the patients except case 1 gave a history of swelling of more than 15 years duration but refused treatment because the lesion was not increasing in size. This was in accordance with Eisenberg and Eisenbud, who revealed that most of the cases of FD cease to progress when skeletal maturity is achieved [17-19, 52, 53].

The development of cystic degeneration as seen in case 2 in FD may result in a diagnostic and therapeutic dilemma. It can be attributed to the fact that it may present clinically as a rapidly enlarging mass which is alarming to both patient and physician [32]. The affected part may lose its anatomical shape and become spherical, thus may appear more cosmetically deformed. Sarcomatous transformation however should always be ruled out in such clinical presentations [47, 54-59].

There is evidence that the lesions of FD may predispose to osteomyelitis [60-62] which necessitates the role of maxillofacial radiology for the better prognosis of the patient [60-62]. Maxillary osteomyelitis occurs rarely in a healthy host and fibro osseous lesions [56] particularly FD [57], is considered as one of the local factors that predispose to this type of infection. Chang *et al.* [58] have described a case of FD with chronic osteomyelitis of mandible. On the contrary, Gupta *et al.* has reported a case of chronic osteomyelitis of mandible which was mimicking FD in the clinical as well as radiological presentation [61].

Henceforth CT scan in best of its ability can help to evaluate the detail of both hard as well as soft tissues of CFD [38, 45, 59]. On the contrary MRI can also provide details about the soft tissues and is considered more sensitive to pathological changes as compared to CT, however it is difficult to interpret in case of FD [38, 45, 59]. It can be attributed to the fact that MRI in FD produces low signal intensity which leads to difficult interpretation [59].

## CONCLUSION:

It can be concluded that advanced imaging modality like CT can provide with exact diagnosis as well as extent of the lesions like FD. Further collaboration of researchers is required to incorporate this proposed change in classification of CFD.

## Figures and Tables

**Fig. (1A):**
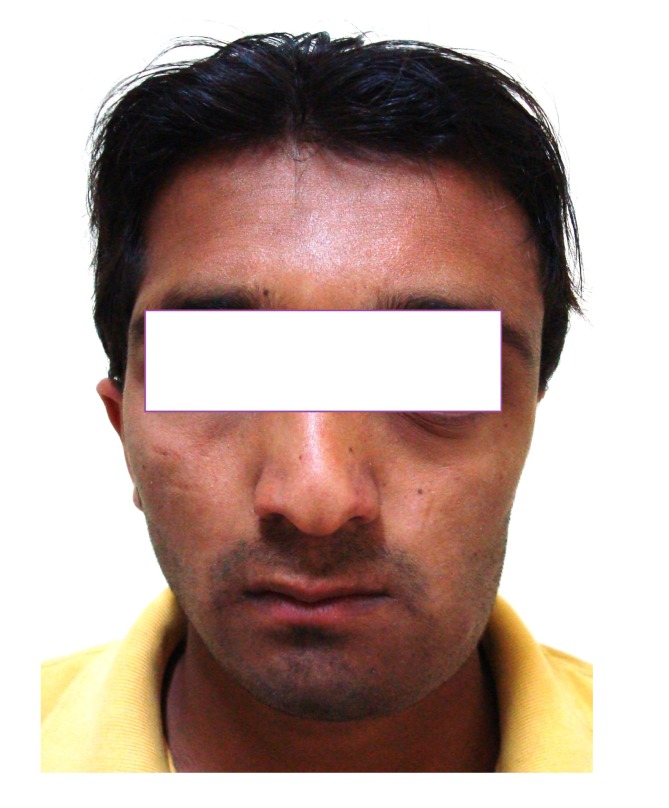
Extra oral profile of the patient with facial asymmetry.

**Fig. (1B):**
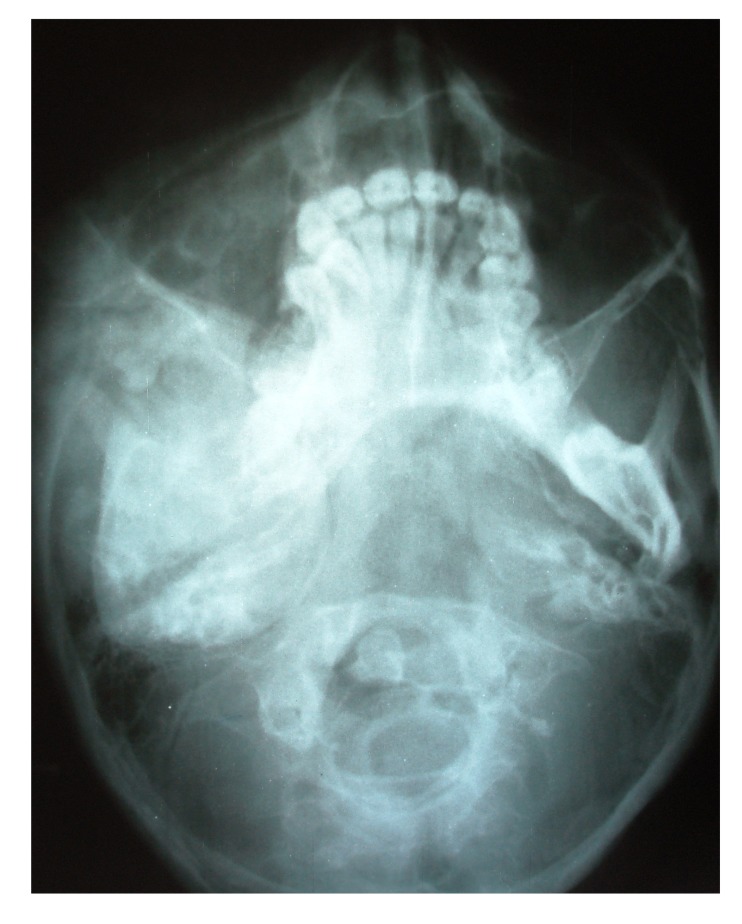
Submentovertex radiograph revealing expansion of the mandible of left side.

**Fig. (1C):**
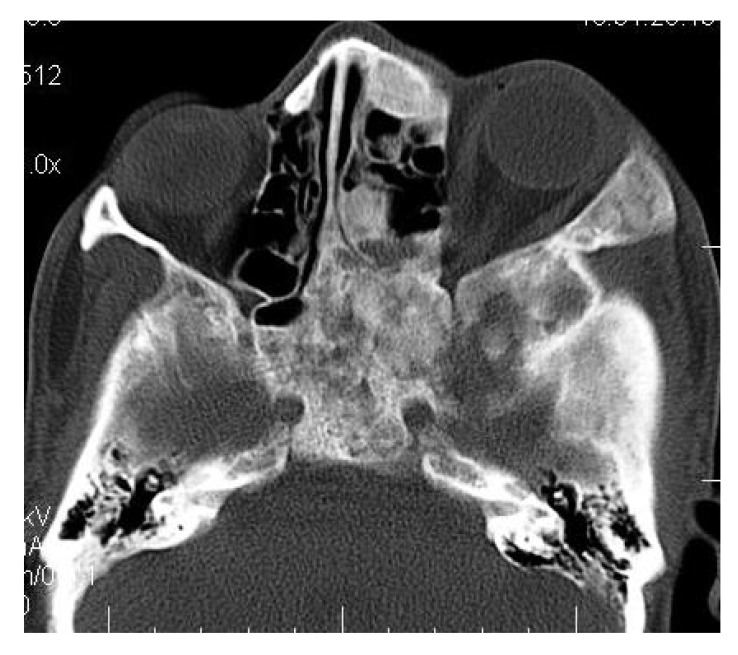
Axial CT slice with bone window revealing involvement of temporal, zygomatic, frontal and ethmoid bones. Sphenoid bone was also involved with narrowing of the optic canal as well as orbital fissures.

**Fig. (1D):**
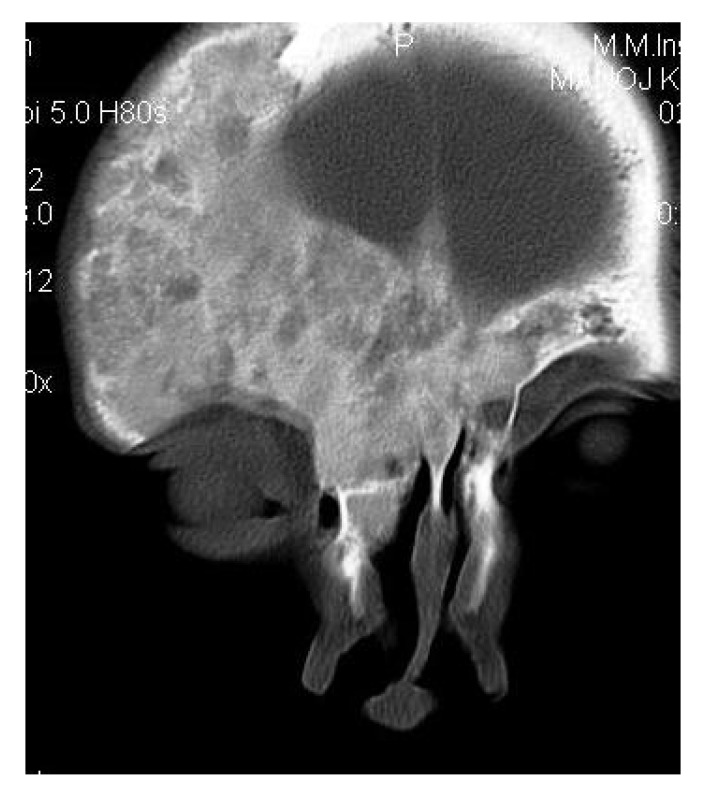
Coronal CT section revealing the lesion crossing the midline in frontal region with involvement of Crista Gali.

**Fig. (1E):**
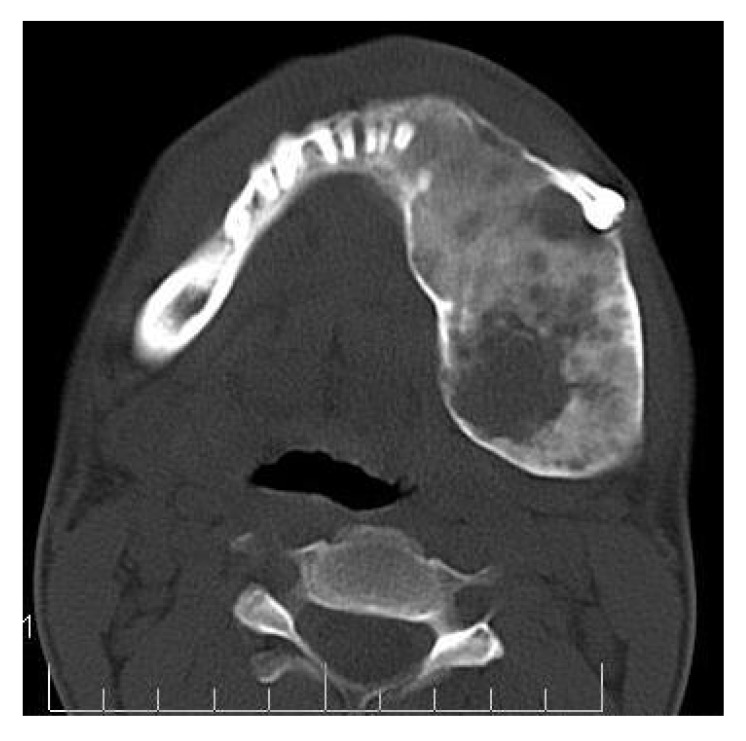
Axial CT section reveals expansion of the mandibular body with erosions and destruction of the internal structures. Tooth displacement was also observed.

**Fig. (2A):**
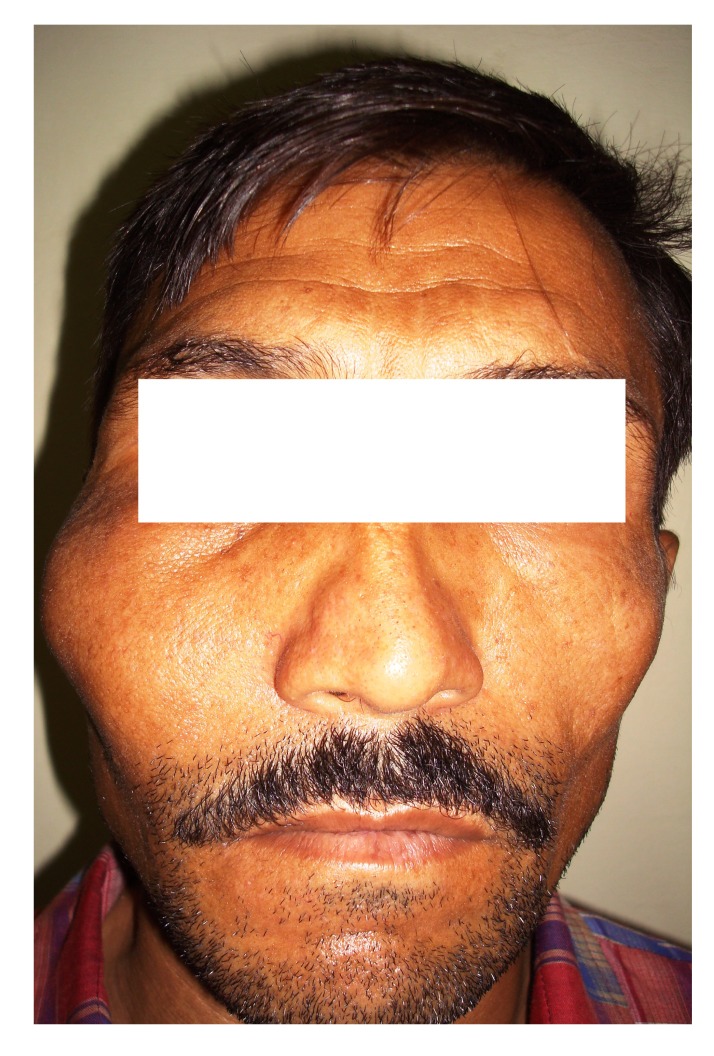
Patient with asymmetry of the maxillofacial region.

**Fig. (2B):**
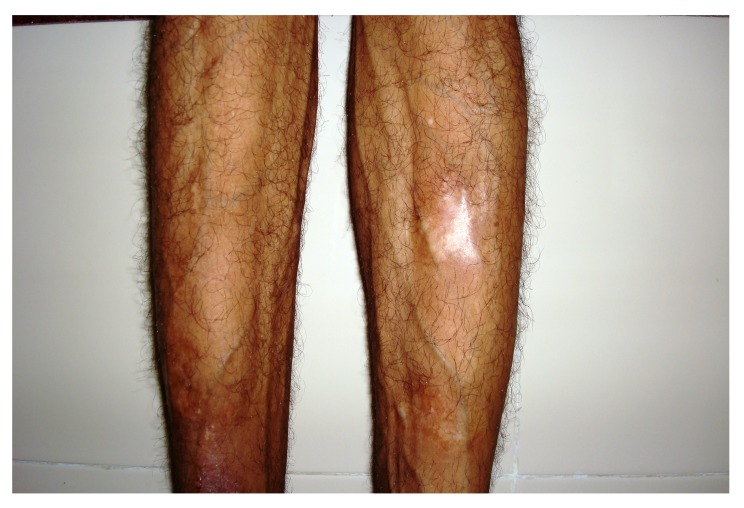
Swelling along the left shin of tibia.

**Fig. (2C):**
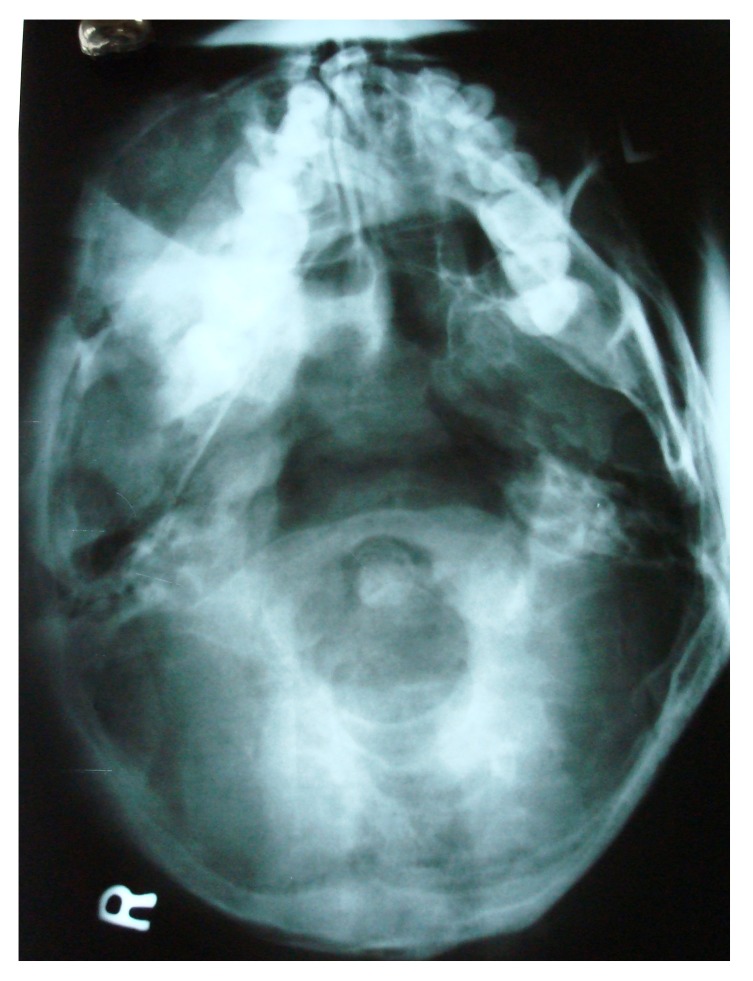
Submentovertex view revealing expansion of the right side of the craniofacial structures including maxilla, mandible and temporal bones.

**Fig. (2D):**
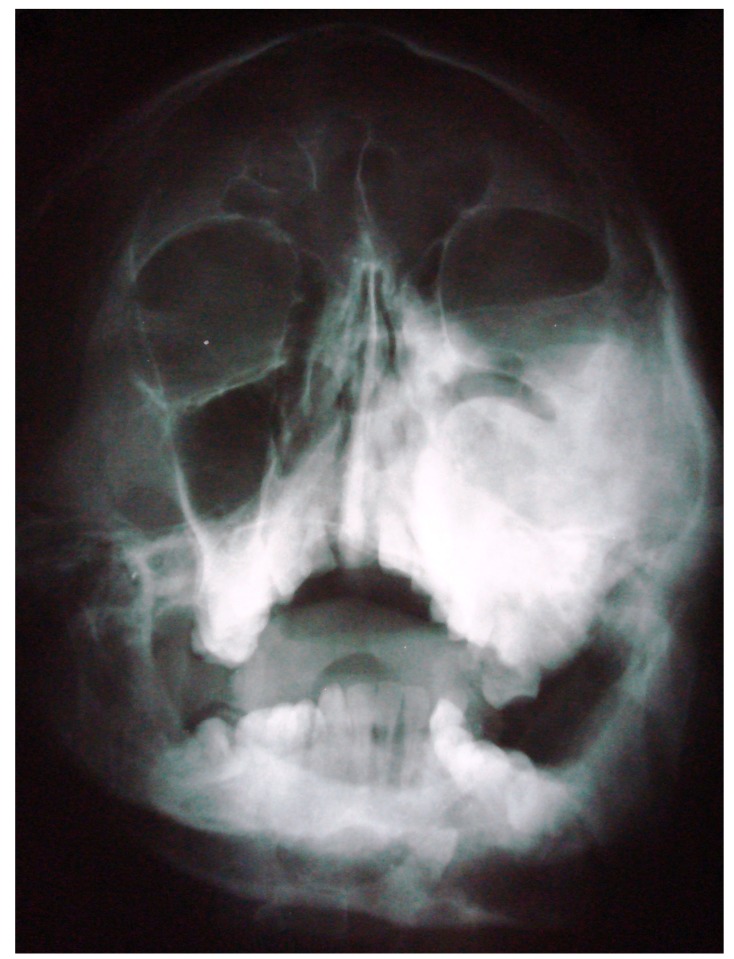
PNS view revealing expansion of the right side of the craniofacial structures including maxilla, mandible and temporal bones.

**Fig. (2E):**
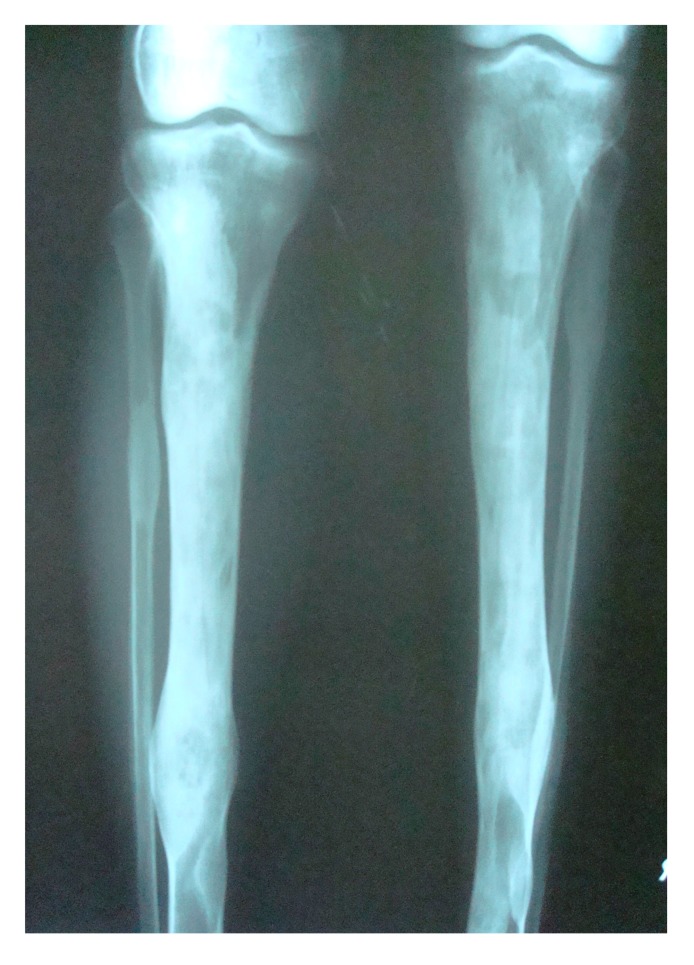
Lateral AP view of Tibia with evidence of sclerosis of upper and mid shaft of tibia along with lytic areas in the sclerotic irregularity of the bone.

**Fig. (2F):**
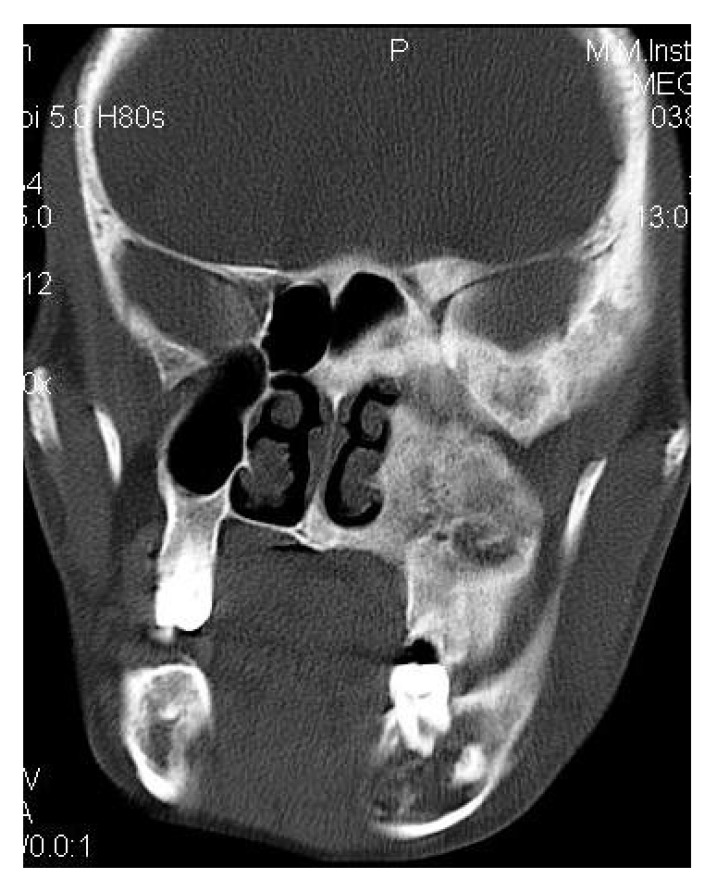
Coronal CT section showing the involvement of right side of mandible with the TMJ, glenoid fossa, temporal bone, frontal bone and maxilla.

**Fig. (2G):**
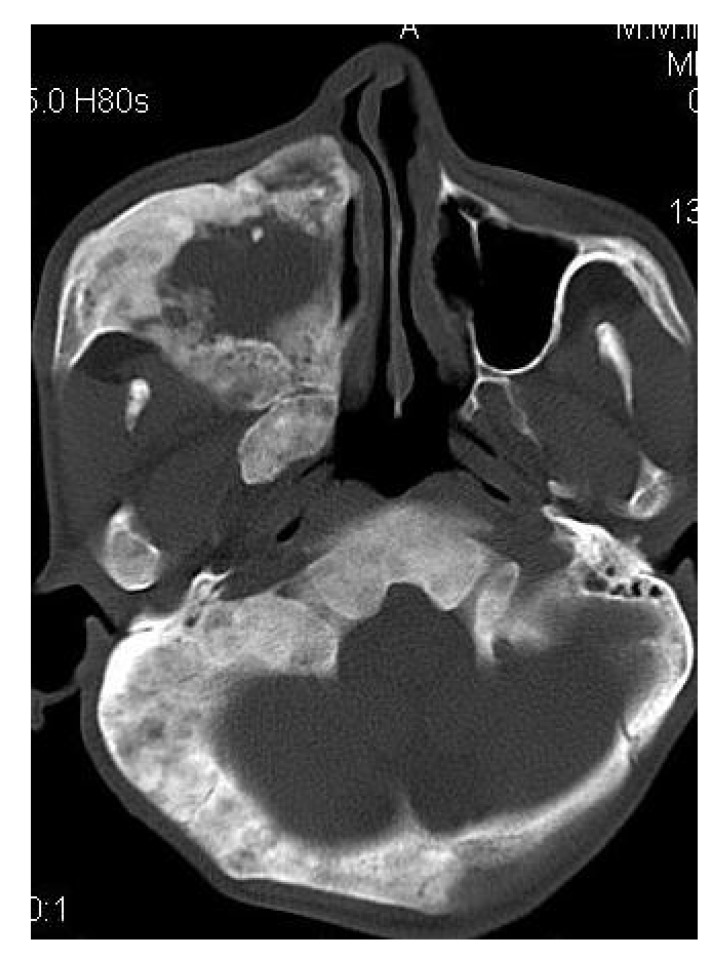
Axial CT section showing the involvement of the maxillary sinus of right side. Further the zygomatic arch, zygoma, base of the cranium, mastoid process, pterygoid plates and sphenoid of right side were also involved. There was evidence of involvement of occipital condyles of both the sides and clivus.

**Fig. (2H):**
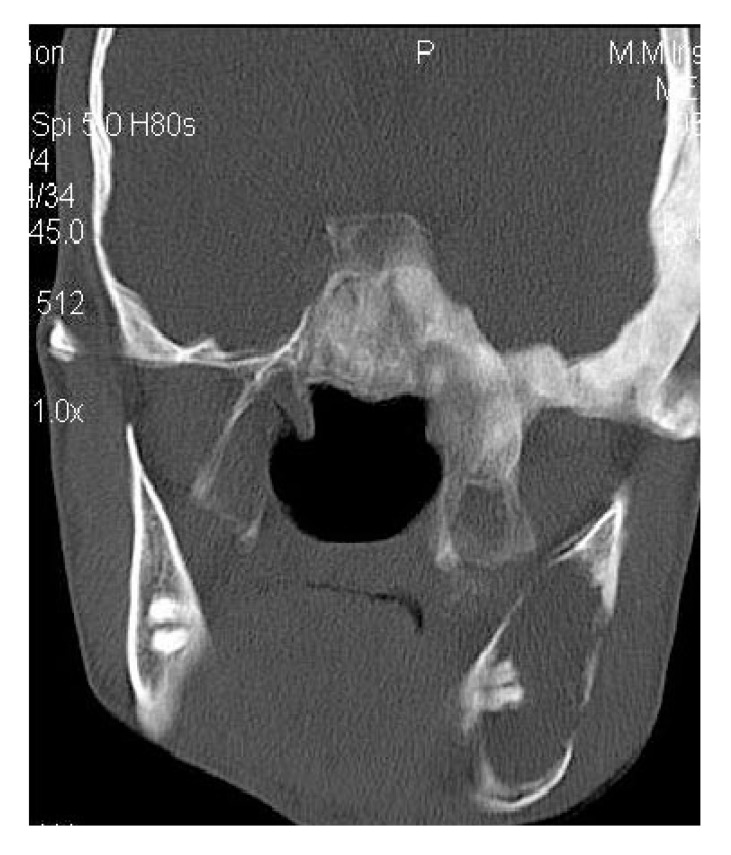
Coronal CT section showing the involvement of the zygomatic arch, zygoma, base of the cranium, mastoid process, pterygoid plates and sphenoid of right side. There was evidence of involvement of occipital condyles of both the sides and clivus with evidence of a cystic lesion in the right mandible extending from body of the mandible to the ramus.

**Fig. (2I):**
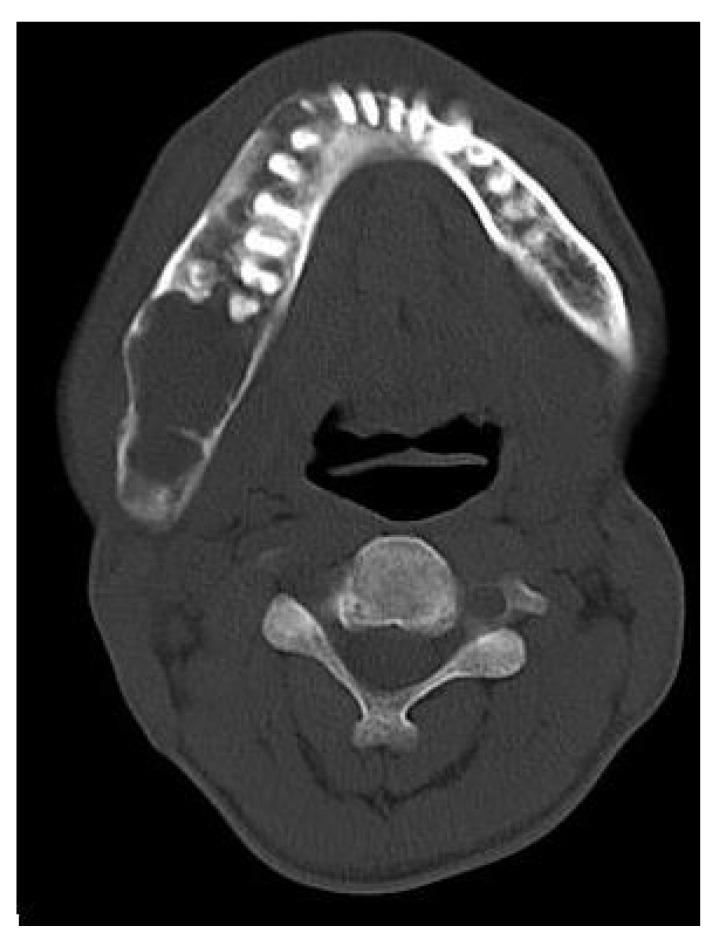
Axial CT section with evidence of a cystic lesion in the right mandible extending from body of the mandible to the ramus of right side.

**Fig. (3A):**
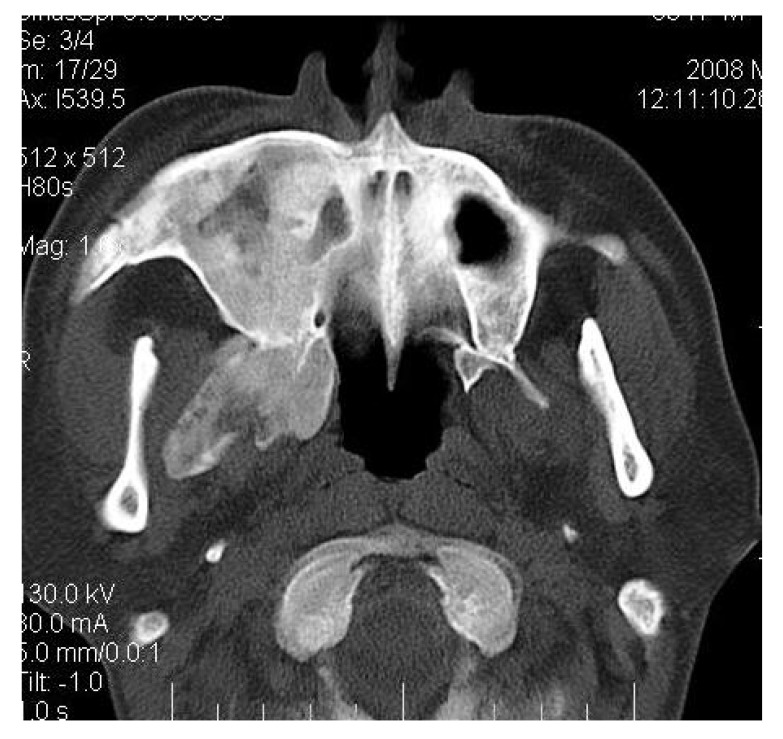
The Axial CT section showing the involvement of right side of the maxilla including the maxillary sinus, right zygoma, zygomatic arch, pterygoid plates.

**Fig. (3B):**
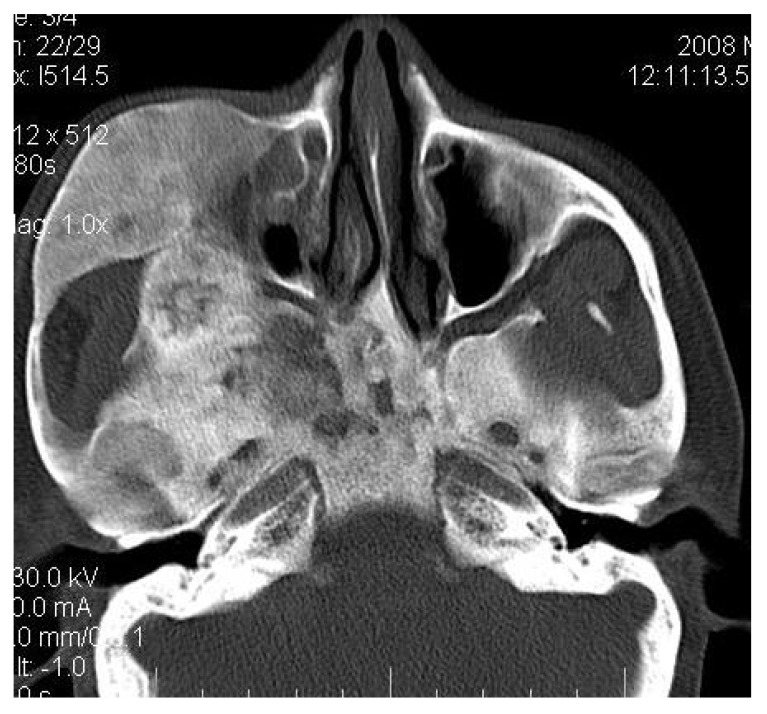
The Axial CT section showing the involvement of right side of the maxillary sinus, right zygoma, zygomatic arch, right sphenoid, pterygoid plates of right side along with the cranial base.

**Fig. (3C):**
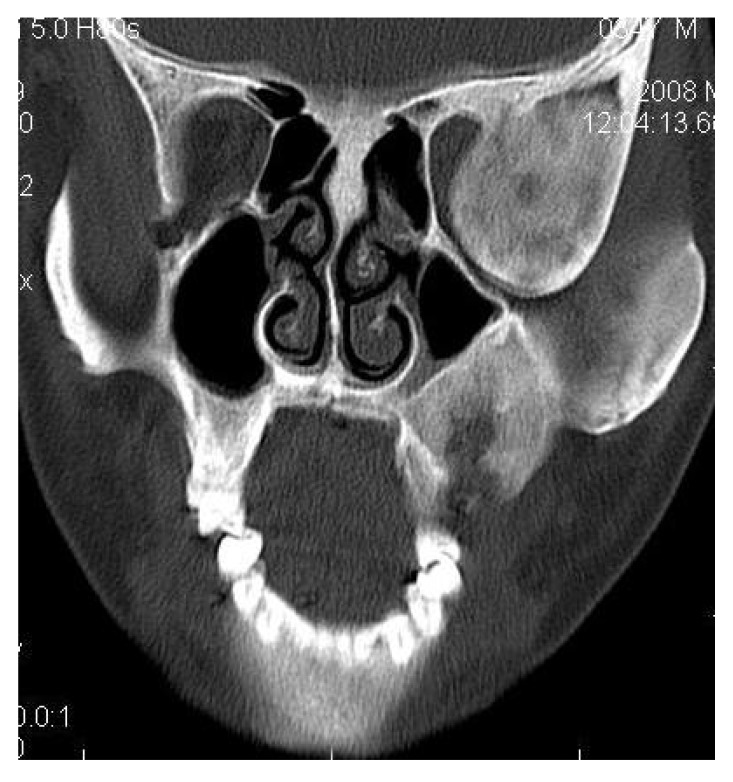
The coronal CT section revealing the involvement of the right orbit, maxilla and right Zygomatic arch and right posterior maxillary alveolar process.

**Fig. (3D):**
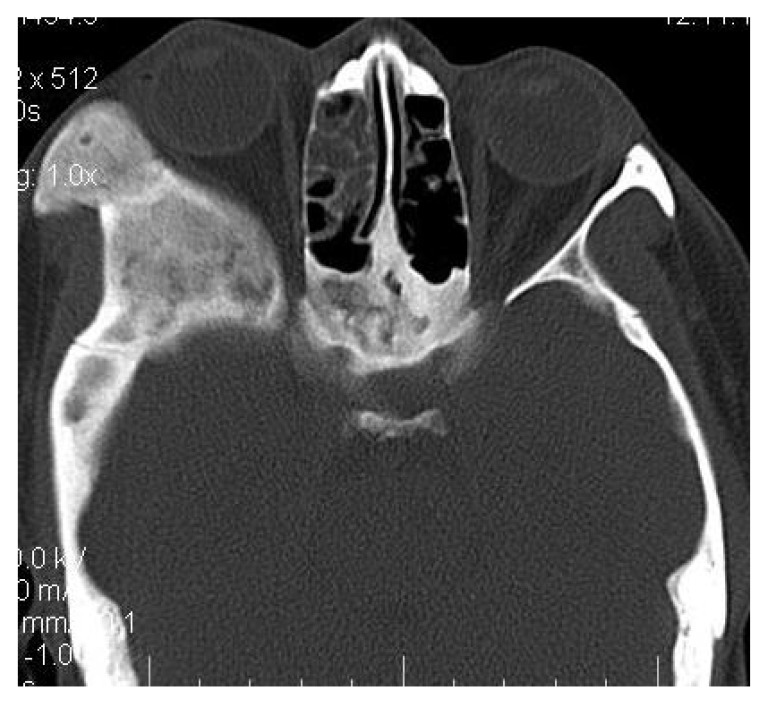
The Axial CT section revealing the involvement of the lateral wall of right orbit, right Zygomatic arch, zygoma, right pterygoid region with right craniaum. Further there was evidence of narrowing of the of the optic canal of right side.

**Fig. (4A):**
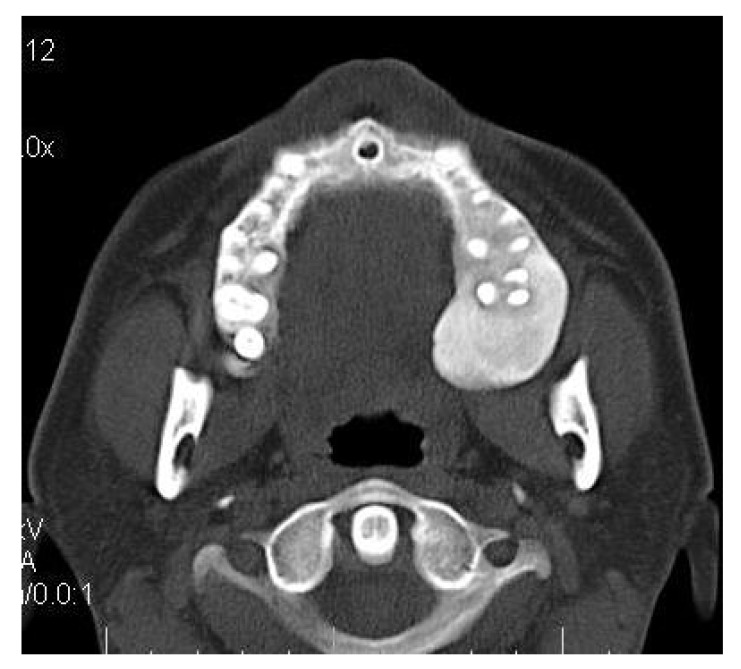
Axial CT section revealing involvement of left maxillary alveolus and tuberosity.

**Fig. (4B):**
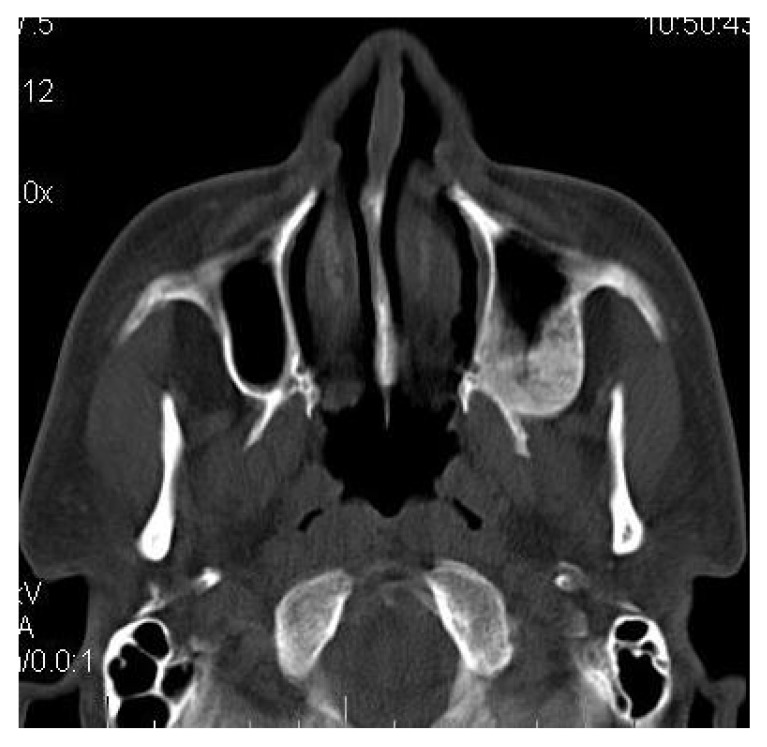
Axial CT section revealing the involvement of the left maxillary sinus and base of the left pterygoid plate.

**Fig. (4C):**
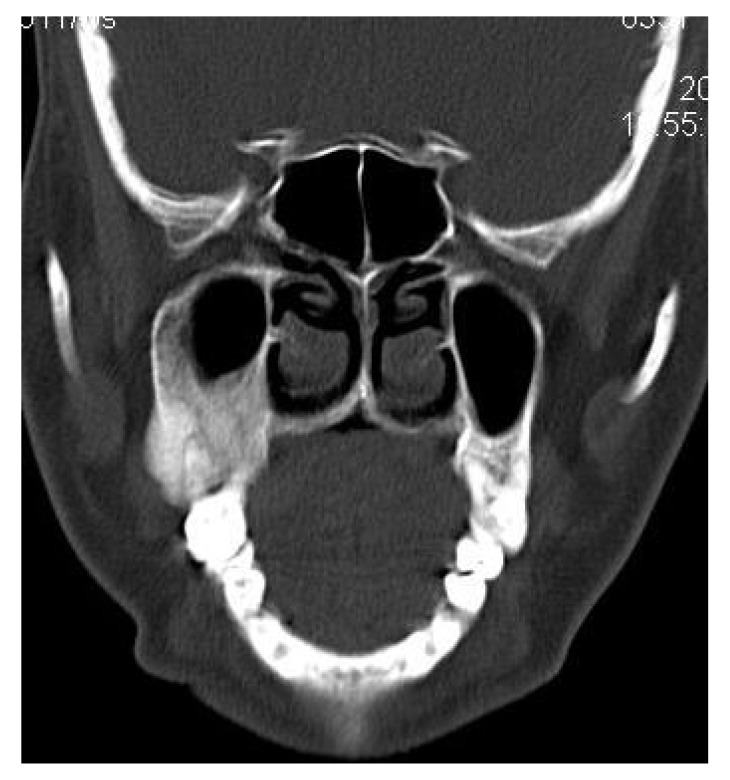
Coronal CT section revealing the involvement of left maxillary sinus and left maxillary alveolus.

**Fig. (4D):**
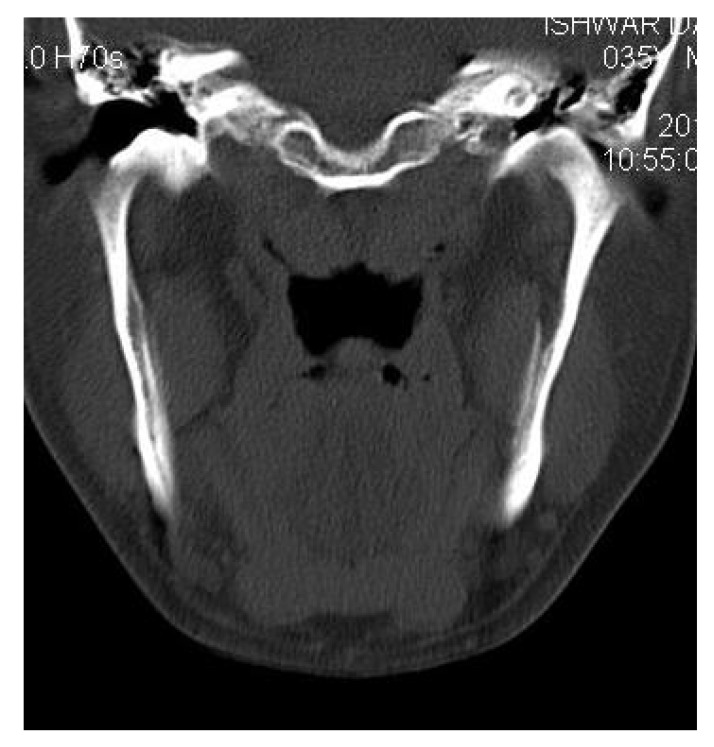
Coronal CT section revealing the normal TMJ.
